# *PERPETUAL FLOWERING2* coordinates the vernalization response and perennial flowering in *Arabis alpina*

**DOI:** 10.1093/jxb/ery423

**Published:** 2018-11-27

**Authors:** Ana Lazaro, Yanhao Zhou, Miriam Giesguth, Kashif Nawaz, Sara Bergonzi, Ales Pecinka, George Coupland, Maria C Albani

**Affiliations:** 1 Botanical Institute, Cologne Biocenter, University of Cologne, Cologne, Germany; 2 Max Planck Institute for Plant Breeding Research, Carl-von-Linné-Weg, Cologne, Germany; 3 Cluster of Excellence on Plant Sciences ‘From Complex Traits towards Synthetic Modules’, Düsseldorf, Germany; 4 Institute of Experimental Botany, Centre of the Region Haná for Biotechnological and Agricultural Research, Šlechtitelů, Olomouc, Czech Republic

**Keywords:** *APETALA2*, *AP2*, *FLOWERING LOCUS C*, *FLC*, juvenility, *PEP1*, *PEP2*, perennial, *PERPETUAL FLOWERING 1*, vernalization

## Abstract

The floral repressor APETALA2 (AP2) in Arabidopsis regulates flowering through the age pathway. The *AP2* ortholog in the alpine perennial *Arabis alpina*, *PERPETUAL FLOWERING 2* (*PEP2*), was previously reported to control flowering through the vernalization pathway via enhancing the expression of another floral repressor *PERPETUAL FLOWERING 1* (*PEP1*), the ortholog of Arabidopsis *FLOWERING LOCUS C* (*FLC*). However, *PEP2* also regulates flowering independently of *PEP1.* To characterize the function of *PEP2*, we analyzed the transcriptomes of *pep2* and *pep1* mutants. The majority of differentially expressed genes were detected between *pep2* and the wild type or between *pep2* and *pep1*, highlighting the importance of the *PEP2* role that is independent of *PEP1*. Here, we demonstrate that *PEP2* activity prevents the up-regulation of the *A. alpina* floral meristem identity genes *FRUITFUL* (*AaFUL*), *LEAFY* (*AaLFY*), and *APETALA1* (*AaAP1*), ensuring floral commitment during vernalization. Young *pep2* seedlings respond to vernalization, suggesting that PEP2 regulates the age-dependent response to vernalization independently of PEP1. The major role of *PEP2* through the *PEP1*-dependent pathway takes place after vernalization, when it facilitates *PEP1* activation both in the main shoot apex and in axillary branches. These multiple roles of *PEP2* in the vernalization response contribute to the *A. alpina* life cycle.

## Teaser

The *Arabis alpina APETALA2* ortholog, *PERPETUAL FLOWERING2*, coordinates the age-dependent response to vernalization and it is required to facilitate the activation of *A. alpina FLOWERING LOCUS C* after vernalization.

## Introduction

Plant adaptation to the environment requires the modification of developmental traits, among which flowering time is key to ensure successful production of offspring. Alpine habitats in which juvenile survival is very low are mainly dominated by perennial species (Billings and Mooney, 1968). In general, the perennial growth habit relies on the differential behavior of meristems on the same plant so that some will stay vegetative whereas others will initiate flowering (Amasino, 2009; Lazaro *et al.*, 2018). The main environmental cue that promotes flowering in alpine species is the exposure to prolonged cold, a process called vernalization. Alpine environments are characterized by short growing seasons and long periods of snow coverage. Thus, to ensure reproductive success, alpine plants initiate flower buds in response to prolonged cold several months or years before anthesis (Diggle, 1997; Meloche and Diggle, 2001). However, exposure to long periods of cold does not always result in flowering. This is especially true for perennial species, as most of them have a prolonged juvenile phase and are not competent to flower at a young age (Bergonzi and Albani, 2011).

The molecular mechanisms regulating flowering in response to vernalization or to the age of the plant have been mainly studied in the annual model plant *Arabidopsis thaliana*. The MADS box transcription factor FLOWERING LOCUS C (FLC) is the major regulator of flowering in response to vernalization (Michaels and Amasino, 1999; Sheldon *et al.*, 2000). FLC transcriptionally regulates floral integrator genes, such as *SUPPRESSOR OF OVEREXPRESSION OF CONSTANS1* (*SOC1*), and genes involved in the age pathway, suggesting an interplay between these two pathways (Deng *et al.*, 2011; Mateos *et al.*, 2017). Comparative studies between Arabidopsis and the alpine perennial *Arabis alpina* demonstrated that the *FLC* ortholog in *A. alpina*, *PERPETUAL FLOWERING1* (*PEP1*), also controls flowering in response to vernalization. In addition, *PEP1* contributes to the perennial growth habit by repressing flowering in a subset of axillary meristems after vernalization (Wang *et al.*, 2009; Lazaro *et al.*, 2018). Flower buds in *A. alpina* are formed during prolonged exposure to vernalizing conditions. The length of vernalization determines *PEP1* reactivation in the inflorescence. After insufficient vernalization, *PEP1* mRNA is reactivated and results in the appearance of floral reversion phenotypes such as bracts and vegetative inflorescence branches (Lazaro *et al.*, 2018). In the axillary branches, the duration of vernalization does not influence *PEP1* expression, and *PEP1* transcript is high irrespective of the duration of vernalization (Lazaro *et al.*, 2018). The fate of these axillary branches is determined by a combined action of the age pathway and *PEP1* (Wang *et al.*, 2011; Park *et al.*, 2017).

In Arabidopsis, the age pathway is regulated by two miRNAs and their targets. At a young age high miRNA 156 (miR156) levels prevent flowering. As plants get older miR156 accumulation gradually decreases, whereas miR172 levels follow the opposite pattern and gradually increase during development (Wu *et al.*, 2009). The biological function of miR156 is exerted by its targets that encode members of the SQUAMOSA PROMOTER BINDING PROTEIN-LIKEs (SPLs) transcription factor family (Schwab *et al.*, 2005; Wu and Poethig, 2006; Wu *et al.*, 2009; Xu *et al.*, 2016). From these, SPL9 and SPL15 have been reported to activate the transcription of *miRNA172b*, which in turn represses the expression of a small subfamily of APETALA2-like transcription factors by a translational mechanism (Aukerman and Sakai, 2003; Chen, 2004; Mathieu *et al.*, 2009; Wu *et al.*, 2009; Hyun *et al.*, 2016). This subfamily includes six members: AP2, TARGET OF EARLY ACTIVATION TAGGED1 to 3 (TOE1–TOE3), SCHLAFMUTZE (SMZ), and SCHNARCHZAPFEN (SNZ) (Aukerman and Sakai, 2003; Schmid *et al.*, 2003; Mathieu *et al.*, 2009; Yant *et al.*, 2010). *Arabis alpina* has a very distinct juvenile phase and the accession Pajares needs to grow for at least 5 weeks in long days (LDs) before it is able to flower in response to vernalization (Wang *et al.*, 2011; Bergonzi *et al.*, 2013). The role of miR156 is conserved in *A. alpina* as *miR156b*-overexpressing lines block flowering in response to vernalization, while mimicry lines (MIM156), used to reduce miRNA activity, flower when vernalized at the age of 3 weeks (Bergonzi *et al.*, 2013). However, the complementary temporal accumulation of miR156 and miR172 during development is uncoupled in *A. alpina* (Bergonzi *et al.*, 2013). Similar to *A. thaliana*, the accumulation of miR156 is reduced in the shoot apex of *A. alpina* plants that get older and acquire competence to flower in long days, but miR172 expression is low (Bergonzi *et al.*, 2013). For flowering to occur and to observe an increase in miR172 levels in the shoot apex, vernalization is required (Bergonzi *et al.*, 2013). However, vernalization is only effective in mature (old) plants but not in juvenile (young) plants that still have high levels of miR156 (Bergonzi *et al.*, 2013). The initiation of flowering during cold in mature plants correlates with the gradual increase in expression of the floral organ identity genes *LEAFY* (*AaLFY*), *FRUITFUL* (*AaFUL*), and *APETALA1* (*AaAP1*) (Lazaro *et al.*, 2018). In perennials, such as apple and poplar, the homologs of the floral repressor *TERMINAL FLOWER1* (*TFL1*) regulate the juvenile period. Transgenic *Malus domestica* and *Populus trichocarpa* lines with decreased expression of *TFL1* have a shortened juvenile phase (Kotoda *et al.*, 2006; Mohamed *et al.*, 2010). Similarly, transgenic *A. alpina* plants in which *AaTFL1* expression was reduced can flower even when they are vernalized at a young age (Wang *et al.*, 2011). Interestingly, these lines can flower after exposure to a short (6 weeks instead of 12 weeks) duration of vernalization. These results again suggest an interplay between the age and the vernalization pathways.

In Arabidopsis, *AP2* influences a variety of developmental processes, including flowering time through the age pathway and floral development (Yant *et al.*, 2010). Strong recessive *ap2* mutant alleles, such as *ap2-12*, flower early in both LDs and short days (SDs) (Yant *et al.*, 2010). Similarly, lesions in the *A. alpina* ortholog of *AP2*, *PEP2*, has been reported to have a flowering time phenotype (Bergonzi *et al.*, 2013). *pep2* mutants flower without vernalization and show compromised perennial traits, similar to *pep1-1* mutant plants (Bergonzi *et al.*, 2013). The effect of *PEP2* on flowering was first related to the vernalization pathway as it promotes the expression of *PEP1* (Bergonzi *et al.*, 2013). In 2-week-old *pep2-1* seedlings, *PEP1* transcript levels are reduced compared with wild-type plants (Bergonzi *et al.*, 2013). However, *PEP2* also has a *PEP1*-independent role in the regulation of flowering time in *A. alpina* as flowering is accelerated in the *pep1-1 pep2-1* double mutant compared with the single mutants (Bergonzi *et al.*, 2013).

Here, we show that during vernalization *PEP2* represses the expression of the floral meristem identity genes *AaFUL*, *AaLFY*, and *AaAP1*. Vernalization accelerates flowering in young *pep2-1* plants, indicating that PEP2 regulates the age-dependent response to vernalization. In addition, we report that the *PEP1*-dependent role of *PEP2* takes place after vernalization because *PEP2* is required to activate *PEP1* after the return to warm temperatures. The involvement of *PEP2* in two different aspects of the vernalization response contributes to the perennial life cycle of *A. alpina*.

## Materials and methods

### Plant material, growth conditions, and phenotyping

The *A. alpina* genotypes used in this study were Pajares (wild type), the *pep2-1* mutant, and the *pep1-1* mutant. The accession Pajares was collected in the Cordillera Cantábrica mountains in Spain at 1400 m altitude (42°59'32''N, 5°45'32''W). Both the *pep2-1* and the *pep1-1* mutant were isolated from an EMS (ethyl methanesulfonate) mutagenesis in the Pajares background (Wang *et al.*, 2009; Bergonzi *et al*., 2013; Nordstrom *et al*., 2013). For the phenotypic analysis, plants were grown in LDs (16 h light and 8 h dark) under temperatures ranging from 20 °C during the day to 18 °C during the night. All vernalization treatments were performed at 4 °C in SD conditions (8 h light and 16 h dark).

Flowering time in the young wild-type, *pep2-1*, and *pep1-1* plants was scored as the number of leaves at flowering and as the number of days to the first open flower after vernalization. Plants were grown for 3 weeks in LD cabinets, vernalized for 12 weeks, and moved back to LDs after cold.

The characterization of flowering time and inflorescence traits with different vernalization durations in the *pep2-1* mutant was performed together with the wild type and the *pep1-1* mutant in an experiment previously published (fig. 6 in Lazaro *et al.*, 2018). The same data for control wild-type plants were used in Lazaro *et al.* (2018). Plants were grown for 5 weeks in a LD greenhouse, vernalized for 8, 12, 18, and 21 weeks, and moved back to LD greenhouse conditions on the same day. Flowering time was measured by recording the date on which the first flower opened after vernalization. The number of flowering and vegetative branches and the number of bracts in the inflorescence were measured at the end of flowering, except for plants vernalized for 8 weeks when the measurements took place 14 weeks after vernalization.

The Arabidopsis genotypes used herein were Columbia-0 (Col-0) wild type, *ap2-7*, and Col *FRI* San Feliu-2 (Sf-2) (Lee and Amasino, 1995). The *ap2-7* mutant was crossed to the Col *FRI* Sf-2, and the *FRI ap2-7* plants were isolated from a selfed F_2_ progeny that showed *ap2* homeotic defects and late flowering.

For the flowering time experiments in Arabidopsis, the total leaf number (rosette and cauline leaves) was scored at the time when the first flower opened.

### Construction of plasmids and plant transformation

To obtain the *ap2-7 PEP2*–VENUS transgenic plant, a 7.4 kb *PEP2* genomic region spanning 4 kb upstream of the translational start and 1195 bp downstream of the translational stop was cloned by PCR (NCBI accession number LT669794.1). Subsequently, the VENUS:9Ala coding sequence was inserted either after the ATG or before the STOP codon of *PEP2* by employing the polymerase incomplete primer extension (PIPE) method (Klock *et al.*, 2008). Primers used for PIPE cloning are summarized in Supplementary Table S1 at *JXB* online. The generated recombinant DNA fragments were integrated in the pEarlyGate301 binary vector and transformed into Col through *Agrobacterium*-mediated floral dip (Clough and Bent, 1998). Selected homozygous lines, Col *ProPEP2::VENUS::PEP2* N6-1-3 and Col *ProPEP2::PEP2::VENUS* C2-1-9, were crossed to *ap2-7*.

### Gene expression analysis

Gene expression analysis was performed on the wild-type, *pep1-1*, and *pep2-1.* For *pep2-1* samples, homozygous plants were selected after genotyping from a segregating population using a cleaved amplified polymorphic (CAP) marker (Forward primer, CAGCTGCACGGTATGTTTTTC; Reverse primer, GCTTTGTCATAAGCCCTGTG) and *Nde*I digestion.

For the analysis of the *PEP1* expression pattern, the wild type and *pep2-1* were grown for 6 weeks in LDs and vernalized for 12 weeks. Main shoot apices were harvested before vernalization, during vernalization, and after vernalization (1, 2, 3, and 4 weeks after the plants returned to warm temperatures). Axillary vegetative apices were harvested from plants growing in LDs 2, 3, and 4 weeks after vernalization. An average of 10 apices were pooled in each sample.

The expression of *PEP1*, *AaSOC1*, *AaFUL*, *AaTFL1*, *AaLFY*, and *AaAP1* transcripts in the young and adult wild type, *pep1-1*, and *pep2-1* was detected in seedlings grown for 3 weeks (young) or 6 weeks (adult) in LDs and vernalized for 12 weeks. Main shoot apices were harvested before vernalization and during cold, at 4, 8, and 12 weeks in vernalization. For the analysis of *AaSPL5*, *AaSPL9*, *AaSPL15*, and miR156, the main shoot apex was harvested from 3-, 4-, and 6-week old wild-type and *pep2-1* plants growing in LDs. An average of 14 apices were pooled in each sample. Expression levels were normalized to both *AaPP2A* and *AaRAN3*, except for miR156 which was normalized to SnoR101.

The expression of *FLC* transcript was analyzed in the shoot apex of *FRI* and *FRI ap2-7* plants grown for 10 d before vernalization, during 40 d of vernalization, and 10 d and 20 d after the return to LD glasshouse conditions. Expression levels were normalized to *UBC21*. Total plant RNA was extracted using the RNeasy Plant Mini Kit (Qiagen), and a DNase treatment was performed with the Ambion DNA-free kit (Invitrogen) to reduce any DNA contamination. Total RNA (1.5 µg) was used to synthesize cDNA through reverse transcription with SuperScript II Reverse Transcriptase (Invitrogen) and oligo dT(18) as primer. A 2 µl aliquot of a cDNA dilution (1:5) was used as the template for each quantitative PCR (qPCR). For the analysis of miR156 and the *SPL*s, total RNA was extracted using the miRNeasy^®^ Mini Kit (Qiagen), and a DNase treatment was performed with the Ambion DNA-Free kit (Invitrogen) to reduce DNA contamination. A 200 ng aliquot of RNA was used for reverse transcription of miR156 and SnoR101 using miR156- and SnoR101-specific primers. qPCRs were performed using a CFX96 and CFX384 Real-Time System (Bio-Rad) and the iQ SYBR Green Supermix detection system. Each data point was derived from two or three independent biological replicates and is shown as the mean ±standard deviation.

Primers used for qPCR for *PEP1*, *AaSOC1*, *AaFUL*, *AaTFL1*, *AaLFY*, *AaAP1*, *AaSPL5*, *AaSPL9*, *AaSPL15*, *AaPP2A*, *AaRAN3*, miR156, and SnoR101 were described previously (Wang *et al.*, 2009, 2011; Bergonzi et al., 2013; Mateos *et al.*, 2017; Lazaro *et al.*, 2018). Primers used for qPCR for *FLC* and *UBC21* were also described elsewhere (Czechowski *et al.*, 2005; Crevillen *et al.*, 2013).

### Statistical analysis

Statistical analyses were performed using the R software. To detect significant differences in gene expression, we controlled for a false discovery rate (FDR) of 0.05 when conducting multiple pairwise comparisons by using Benjamini–Hochberg-corrected *P*-values. Treatments with significant differences are depicted with letters or asterisks. For the *pep2-1* physiological analysis, we conducted multiple pairwise Bonferroni tests (α=0.05) to detect significant differences between the wild type and *pep2-1*. Here, a non-parametric test could not be conducted due to ties created during rank assignment.

### RNAseq analysis

For differential gene expression analysis, we used the RNA sequencing (RNAseq) method on apices from the 3-week-old wild type, and the *pep2-1* and *pep1-1* mutants. *pep2-1* homozygous plants were genotyped from a segregating population using the CAP marker described above. RNA was isolated as described above and total RNA integrity was confirmed on the Agilent BioAnalyzer. The library preparation and sequencing were performed at the Max Planck Genome Center Cologne, Germany (https://mpgc.mpipz.mpg.de/home/). RNAseq was performed with three biological replicates per sample. The libraries were prepared from 1 mg of total RNA using the TruSeq RNA kit (Illumina) and 100 bp single-end reads were sequenced on HiSeq2500 (Illumina). Reads from all samples were mapped on the *A. alpina* reference genome (Willing *et al.*, 2015) using TopHat (Trapnell *et al.*, 2009) with default parameters. Afterwards, CuffDiff (Trapnell *et al.*, 2010) was used to estimate the mRNA level of each gene by calculating fragments per kilobase of exon model per million reads mapped (FPKM). To calculate the differential gene expression among the samples, FPKM values were used. A log_2_ fold change (L_2_FC) ≥1 for up-regulated genes and L_2_FC ≤ –1 for down-regulated genes, both with a *q*-value (adjusted *P*-value) ≤0.05, was used for further analysis.

Gene Ontology (GO) enrichment was performed with the BiNGO plug-in (Maere *et al.*, 2005) implemented in Cytoscape V3.5.1 (Cline *et al.*, 2007). A hypergeometric test was applied to determine the enriched genes, and the Benjamini–Hochberg FDR correction (Benjamini and Hochberg, 1995) was performed in order to limit the number of false positives. The FDR was set up to 0.05.

Sequencing data from this study have been deposited in the Gene Expression Omnibus (GEO) under accession number GSE117977. Sequences of genes studied can be found in the GenBank/EMBL databases under the following accession numbers: *PEP2* (AALP_AA7G245300), cDNA of *PEP1* (FJ755930), coding sequence of *AaLFY* (JF436956), coding sequence of *AaSOC1* (JF436957), *AaAP1* (KFK41337.1), coding sequence of *AaTFL1* (JF436953), and *AaFUL* (KFK27856.1).

## Results

### *PEP2* influences the expression of genes involved in many plant physiological and developmental responses including flowering

**Fig. 1.**
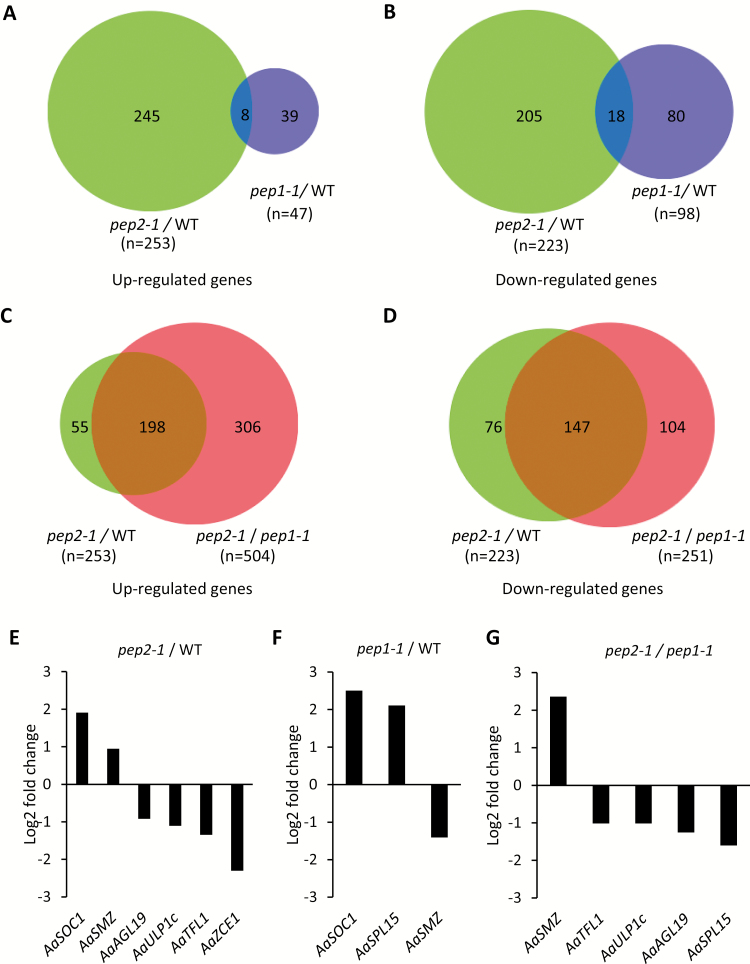
Differentially expressed genes in *pep2* and *pep1 A. alpina* mutants. (A and B) Venn diagram of significantly up-regulated (A) and down-regulated (B) genes in *pep2-1* compared with the wild type (WT) and *pep1-1* compared with the WT. (C and D) Venn diagram of significantly up-regulated (C) and down-regulated (D) genes in *pep2-1* compared with the WT and in *pep2-1* compared with *pep1-1.* (E–G) Flowering time genes differentially expressed in *pep2-1* compared with the WT (E), *pep1-1* compared with the WT (F), and *pep2-1* compared with *pep1-1* (G). Expression values are based on RNAseq.

To provide an overview of the role of *PEP2* in *A. alpina*, we performed an RNAseq analysis. We compared the transcriptomes of apices of 3-week-old *pep2-1* and *pep1-1* mutants with the wild type (Pajares). Three-week-old wild-type and mutant plants are vegetative and have not undergone the transition to flowering (Wang *et al.*, 2011; Bergonzi *et al.*, 2013; Park *et al.*, 2017; Lazaro *et al.*, 2018). Among transcriptomes, the majority of differentially expressed genes were detected in *pep2-1*. A total of 253 genes were up-regulated and 223 genes were down-regulated in *pep2-1* compared with the wild type (Fig. 1A, B; Supplementary Dataset S1). In contrast, only 47 genes were up-regulated and 98 genes were down-regulated in *pep1-1* compared with the wild type (Fig. 1A, B; Supplementary Dataset S2). The genes differentially expressed between *pep1-1* and the wild type are influenced by *PEP1*, whereas those differentially expressed between *pep2-1* and the wild type are affected by *PEP2* through both the *PEP1*-dependent and *PEP1*-independent pathway. To identify genes influenced by *PEP2* through the *PEP1*-independent pathway, we compared the transcriptomes of *pep2-1* versus *pep1-1* (Fig. 1C, D; Supplementary Dataset S3). A total of 504 genes were significantly up-regulated and 251 genes were significantly down-regulated in *pep2-1* compared with *pep1-1* (Fig. 1C, D). Interestingly, the number of differentially expressed genes detected between *pep2-1* and *pep1-1* was higher than those detected when single mutants were compared with the wild type. GO analysis demonstrated that the most enriched category for the up-regulated genes in *pep2-1* compared with the wild type and in *pep2-1* compared with *pep1-1* was the biosynthesis of glucosinolates, which are involved in defense against herbivore attack and pathogens (Supplementary Fig. S1) (Keith and Mitchell-Olds, 2017). The overlap in over-represented GO categories in the set of genes up-regulated in *pep2-1* in comparison with either the wild type or the *pep1-1* mutant was very high, which is to be expected as more genes were up-regulated in *pep2-1* compared with the wild type than in *pep1-1* compared with the wild type (Fig. 1A; Supplementary Fig. S1A, B). Among the commonly enriched categories for down-regulated genes in *pep2-1*, we found apoptosis and protein desumoylation (Supplementary Fig. S1C, D).

Floral activators and repressors were identified among the differentially expressed genes in *pep2-1*. For example, the *A. alpina* ortholog of *SOC1* (*AaSOC1*) was up-regulated in *pep2-1* compared with the wild type (Fig. 1E; Supplementary Dataset S1). This effect of *PEP2* on *AaSOC1* is through *PEP1* as *AaSOC1* was differentially expressed between *pep1-1* and the wild type, but not in *pep2-1* versus *pep1-1* (Fig. 1F, G; Supplementary Datasets S2, S3; Mateos *et al*, 2017). The regulation of *AaSMZ* through *PEP2* is different from that through *PEP1*. *AaSMZ* was up-regulated in *pep2-1* compared with the wild type and down-regulated in *pep1-1* compared with the wild type (Fig. 1E, F; Supplementary Datasets S1, S2). In contrast, *AaSPL15* was up-regulated in the *pep1-1* mutant compared with the wild type and not in *pep2-1* compared with the wild type, indicating that *PEP2* does not control *AaSPL15* expression (Fig. 1E, F; Supplementary Datasets S1, S2). Among the flowering time genes involved in the *PEP1*-independent role of *PEP2* were the floral repressor *AaTFL1* and *AGAMOUS-LIKE 19* (*AaAGL19*). *AaTFL1* was down-regulated when we compared *pep2-1* with both the wild type and *pep1-1*, suggesting that the effect of *PEP2* on *AaTFL1* is independent of *PEP1* (Fig. 1E–G; Supplementary Datasets S1–S3). Similarly, *AGAMOUS-LIKE 19* (*AaAGL19*) transcripts were down-regulated specifically in the *pep2-1* mutant (Fig. 1E–G; Supplementary Datasets S1–S3). We also found the ortholog of *AaULP1c* (ubiquitin-like protein protease) encoding for a SUMO protease and of *CIS-CINNAMIC ACID-ENHANCED 1* (*AaZCE1*) being differentially expressed specifically in *pep2-1* (Fig. 1C–E; Supplementary Datasets S1, S2). Interestingly, both *ULP1c* and *ZCE1* in Arabidopsis control flowering through *FLC*. Mutations in *ULP1c* and its homolog, *ULP1d*, in Arabidopsis cause an early flowering phenotype that can at least partially be due to *FLC* down-regulation (Conti *et al.*, 2008; Castro *et al.*, 2016). *ZCE1* is involved in the regulation of plant growth and development by *cis*-phenylpropanoids and it has been shown to control bolting time via enhancing *FLC* expression (Guo *et al.*, 2011).

### *PEP2* can complement the Arabidopsis *ap2* mutant

**Fig. 2.**
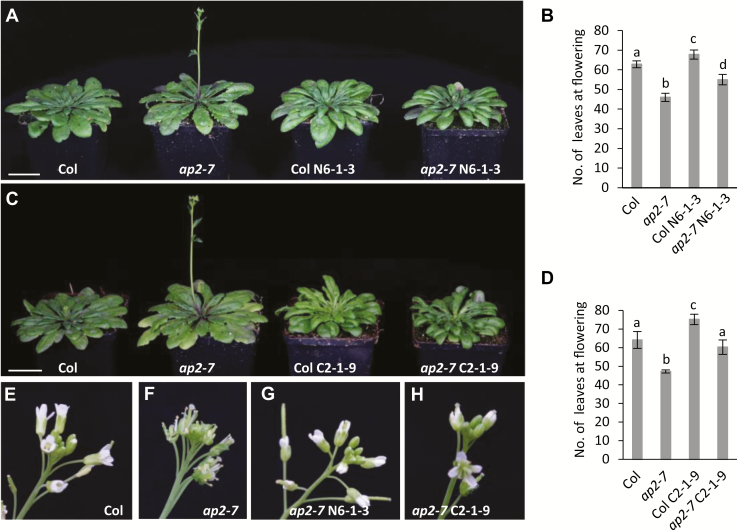
*PEP2* can complement the flowering and floral phenotype of the Arabidopsis *ap2-7* mutant. (A and B) Phenotypes of Col wild type, the *ap2-7* mutant, the Col *ProPEP2::VENUS::PEP2* N6-1-3, and the *ap2-7 ProPEP2::VENUS::PEP2* N6-1-3 lines grown in SDs (A) and number of leaves at flowering (B). (C and D) Col, the *ap2-7* mutant, the Col *ProPEP2::PEP2::VENUS* C2-1-9, and the *ap2-7 ProPEP2::PEP2::VENUS* C2-1-9 lines grown in SDs (C) and number of leaves at flowering (D). (A and C) Whole plant pictures were taken 57 days after germination (DAG). Scale bar=3 cm. In (B) and (D), letters stand for significant differences between genotypes determined by multiple pairwise comparisons using Benjamini–Hochberg-corrected *P*-values (a-value of 0.05). Error bars indicate the standard deviation. (E and F) Inflorescence of Col wild type (E) *ap2-7* (F), *ap2-7 ProPEP2::VENUS::PEP2* N6-1-3 (G), and *ap2-7 ProPEP2::PEP2::VENUS* C2-1-9 (H) taken 73 DAG in SDs.

Both the *pep2* mutant in *A. alpina* and the *ap2* mutant in Arabidopsis show early flowering and similar floral defects, including the absence of petals and the transformation of sepals to carpels (Bowman *et al.*, 1991; Bergonzi et al., 2013; Nördstrom *et al.*, 2013). To check if both genes have common functions, we expressed *PEP2* in the *ap2-7* mutant background under the control of its own promoter. We fused a 7.4 kb *PEP2* genomic region spanning 4 kb upstream of the translational start and 1.2 kb downstream of the translational stop to the VENUS fluorescent protein, at the N- or C-terminus. Transgenic lines were first obtained in the Col background. Homozygous lines obtained for the N-terminal VENUS (Col *ProPEP2::VENUS::PEP2* N6-1-3) and the C-terminal VENUS (Col *ProPEP2::PEP2::VENUS* C2-1-9) were subsequently crossed to *ap2-7*. When grown in SDs, the *PEP2* constructs complemented the early flowering phenotype of the *ap2-7* mutant (Fig. 2A–D). Moreover, the homeotic defects of the *ap2* mutant were restored by *PEP2*, indicating that the *A. alpina PEP2* gene controls flowering time and floral organ identity in a similar way to *AP2* (Fig. 2E–H).

To test whether the effect of *PEP2* on *PEP1* expression was conserved in Arabidopsis for *AP2* and *FLC*, we combined the *ap2-7* mutation with the strong *FRI* allele from the San Feliu-2 (Sf-2) accession, which enhances Col *FLC* expression. Although the *ap2* mutation accelerated flowering in the *FRI* Sf-2 background, the expression of *FLC* was not altered in the apices of these plants at different developmental stages (before, during, or after 40 d of vernalization) (Supplementary Fig. S2). These results indicate that, although the role of *AP2* and *PEP2* regarding flowering time regulation and floral organ identity is conserved, *AP2* does not control *FLC* expression in a *FRI* Sf-2 background (Supplementary Fig. S2B).

### *PEP2* controls the age-dependent response of *A. alpina* to vernalization

**Fig. 3.**
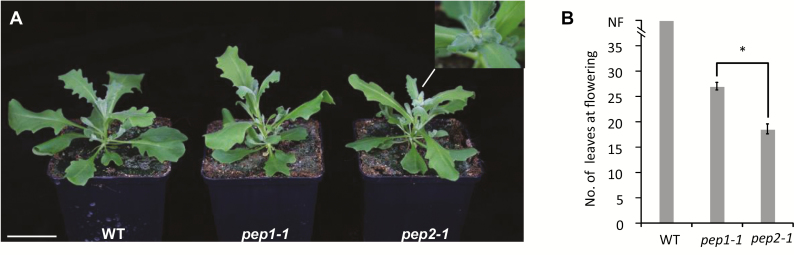
*PEP2* regulates the age-dependent response of *A. alpina* to vernalization. (A) Picture of 3-week-old wild-type (WT), *pep1-1*, and *pep2-1* vernalized for 12 weeks followed by 2 weeks in LDs. Scale bar=5 cm. (B) Flowering time demonstrated as the number of leaves at flowering of 3-week-old WT, *pep1-1*, and *pep2-1* vernalized for 12 weeks. The WT did not flower (NF). The asterisk stands for a significant difference in the total leaf number determined by a Student *t*-test (*P*-value <0.01). Error bars indicate the standard deviation.

We then investigated whether the *PEP1*-independent role of *PEP2* was similar to that of *AP2* in Arabidopsis and, therefore, whether it regulated flowering through the age pathway. We first analyzed the accumulation of miR156 and the transcript level of the *A. alpina SPL5*, *9*, and *15* (*AaSPL5, 9*, and *15*) in the apices of *pep2-1* and wild-type seedlings grown for 3, 4, and 6 weeks in LDs (Supplementary Fig. S3). miR156 accumulation in the shoot apex decreased in older seedlings, but a similar pattern was observed in *pep2-1* and the wild type (Supplementary Fig. S3A). Transcript levels of *AaSPL5*, *9*, and *15* increased as the plants aged (Supplementary Fig. S3B–D). For *AaSPL5* and *15*, we observed no significant differences between *pep2-1* and the wild type, whereas *AaSPL9* mRNA levels differed between the two genotypes only in 6-week-old seedlings (Supplementary Fig. S3B–D). These results are consistent with previous studies in Arabidopsis demonstrating that *AP2* controls flowering through the age pathway downstream of miR156 and the *SPL* genes. As it was previously shown that the age-dependent effect on flowering in *A. alpina* is only apparent after vernalization (Wang *et al.*, 2011; Bergonzi *et al.*, 2013), we tested whether *PEP2* has an age-dependent role in vernalized plants. For this, we vernalized 3-week-old wild-type and *pep2-1* seedlings for 12 weeks and measured flowering time after the return to warm temperatures. We also included the *pep1-1* mutant in this experiment to rule out a *PEP1*-dependent effect of *PEP2* on flowering time. In accordance with previous studies, under these conditions the wild type did not flower after vernalization and only grew vegetatively (Fig. 3; Wang *et al.*, 2011; Bergonzi *et al*., 2013). Interestingly, *pep2-1* flowered with an average of 18 leaves and 17 d after vernalization, whereas vernalized *pep1-1* flowered with 27 leaves similar to non-vernalized *pep1-1* plants grown continuously in LDs (Fig. 3; Supplementary Fig. S4; Wang *et al.*, 2009; Bergonzi *et al.*, 2013). These data suggest that vernalization accelerates flowering in young *pep2-1* but not in *pep1-1* plants. The flowering time phenotype of the mutants is also in contrast to that in LDs when *pep1-1* flowers earlier than *pep2-1* (Bergonzi *et al.*, 2013). Overall, these results suggest that *PEP2* regulates the age-dependent response to vernalization in a *PEP1*-independent manner.

**Fig. 4.**
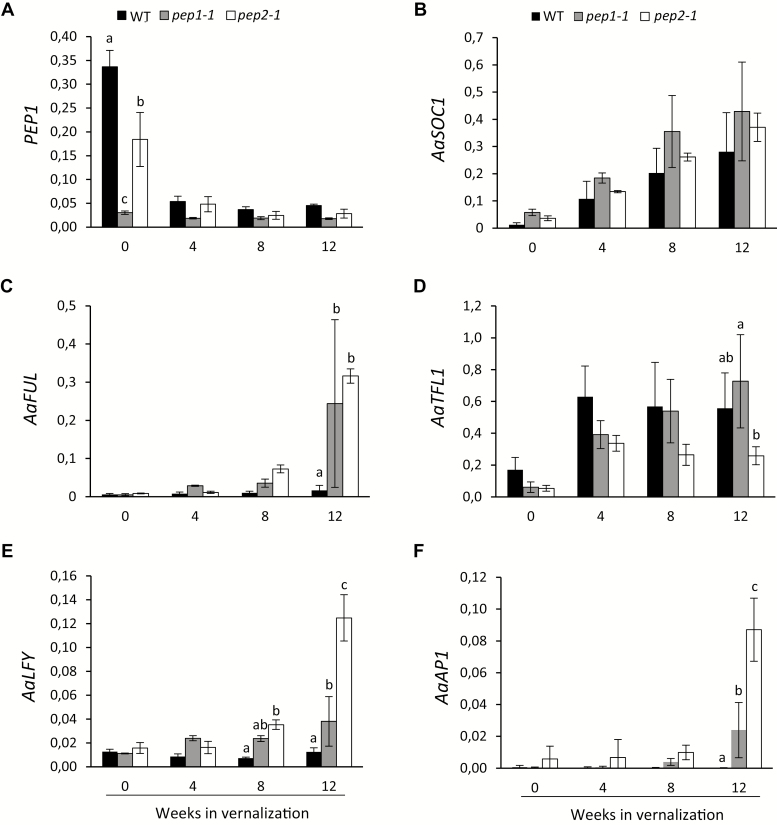
*PEP2* regulates *AaFUL*, *AaTFL1*, *AaLFY*, and *AaAP1* expression during vernalization. Relative expression of *PEP1* (A), *AaSOC1* (B), *AaFUL* (C), *AaTFL1* (D), *AaLFY* (E), and *AaAP1* (F). Three-week-old wild-type (WT), *pep1-1*, and *pep2-1* shoot apices were harvested before and during 12 weeks of vernalization. Letters stand for significant differences between the WT, *pep1-1*, and *pep2-1* at each time point determined by multiple pairwise comparisons using Benjamini–Hochberg-corrected *P*-values (α-value of 0.05). Graphs with no letters show no significant differences. Error bars indicate the standard deviation.

To understand how the young *pep2-1* plants accelerate flowering in response to vernalization, we analyzed the expression of *PEP1*, *AaSOC1*, *AaFUL*, *AaTFL1*, *AaLFY*, and *AaAP1*. Three-week-old wild-type, *pep2-1*, and *pep1-1* apices from the main shoot were harvested before and during vernalization at 4, 8, and 12 weeks. In agreement with previous results obtained in seedlings, non-vernalized 3-week-old *pep2-1* plants showed lower *PEP1* mRNA levels than the wild type (Fig. 4A; Bergonzi *et al.*, 2013). Nevertheless, the *PEP1* transcript decreased in a similar way in *pep2-1* and wild-type plants, and *PEP1* was silenced after 4 weeks in cold (Fig. 4A). These data suggest that, despite the initial difference in *PEP1* expression, the lack of *PEP2* does not influence *PEP1* transcription in young apices during vernalization. The expression of *AaSOC1* was gradually up-regulated during vernalization, following the same pattern in the three genotypes (Fig. 4B). In contrast, *AaFUL*, *AaLFY*, and *AaAP1* showed a differential increase in the wild type and the mutants after 8 and 12 weeks in vernalization (Fig. 4C, E, F). In young wild-type plants *AaFUL*, *AaLFY*, and *AaAP1* mRNA levels did not rise, indicating that flowering had not been initiated (Fig. 4C, E, F). Moreover, the *pep2-1* mutant showed higher levels of *AaLFY* and *AaAP1* than *pep1-1* after 12 weeks in vernalization (Figs 3, 4E, F). Interestingly, the *pep2-1* mutant also showed reduced expression of *AaTFL1* at the end of the cold treatment compared with *pep1-1* (Fig. 4D). Taken together, our results indicate that *PEP2* activates *AaTFL1* and represses *AaFUL*, *AaLFY*, and *AaAP1* in young apices during vernalization (Fig. 3). This role of *PEP2* is independent of *PEP1*, given that *pep1-1* plants vernalized at a young age flowered later than *pep2-1* and that *PEP1* expression was reduced to the same extent in the wild type and *pep2-1* during vernalization (Figs 3, 4A).

To investigate whether the regulation of these floral meristem identity genes by *PEP2* was also conserved in adult plants, we tested the expression of *AaFUL*, *AaLFY*, and *AaAP1* during vernalization. Six-week-old wild-type and *pep2-1* plants were exposed to 12 weeks of cold and the mRNA levels of *AaLFY*, *AaAP1*, and *AaFUL* were analyzed in the shoot apex before vernalization and 1, 3, 5, 8, and 12 weeks into vernalization. *AaFUL* mRNA levels were higher in *pep2-1* than in the wild type already after 8 weeks of vernalization (Supplementary Fig. S4A). For *AaLFY* and *AaAP1* expression, a significant increase was observed in the *pep2-1* mutant only at the end of the 12 weeks of cold (Supplementary Fig. S4B, C). Overall, these results suggest that *PEP2* delays flowering by keeping *AaFUL*, *AaLFY*, and *AaAP1* repressed at the end of the 12 weeks of vernalization, when *PEP1* has already been silenced in the apices of both young and adult plants.

### *PEP2* is required to activate *PEP1* expression after vernalization

**Fig. 5.**
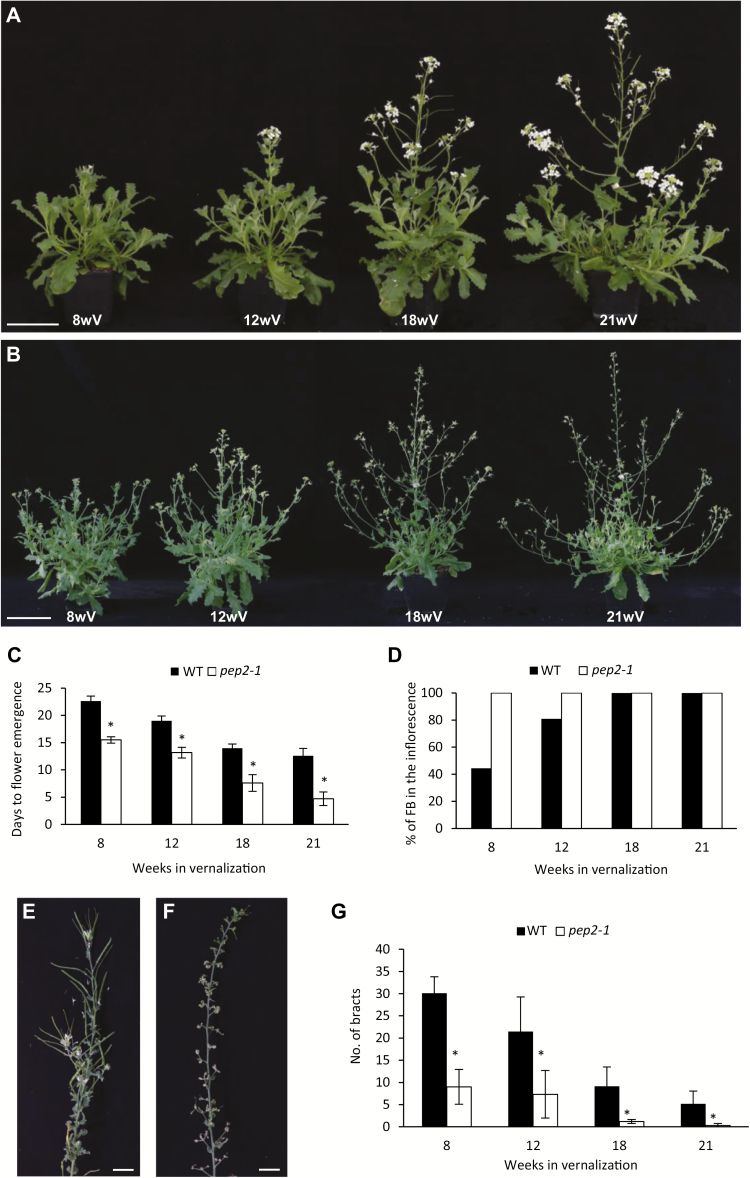
The *pep2* mutant plants flower earlier than the wild type and show reduced reverted phenotypes. (A) Wild-type (WT) plants exposed to several durations of vernalization (8, 12, 18, and 21 weeks) followed by 3 weeks in LDs. (B) *pep2-1* mutant plants exposed to several durations of vernalization (8, 12, 18, and 21 weeks) followed by 3 weeks in LDs. Scale bar=10 cm. (C) Time to flower emergence of WT and *pep2-1* plants exposed to different durations of vernalization measured as the number of days to the first open flower. (D) Percentage of flowering inflorescence branches (FB) in the WT and the *pep2-1* mutant exposed to 8, 12, 18, and 21 weeks of vernalization at the time the last flower in the inflorescence opened. (E) WT reverted inflorescence in plants vernalized for 8 weeks. (F) *pep2-1* mutant inflorescence in plants vernalized for 8 weeks. Scale bar=2 cm. (G) Number of bracts within the inflorescence of the WT and the *pep2-1* mutant exposed to 8, 12, 18, and 21 weeks of vernalization at the time the last flower in the inflorescence opened. This experiment was performed together with the *pep1-1* mutant in an experiment previously published (Fig. 6 in Lazaro *et al.*, 2018). Data for the WT control is similar between the two papers. Asterisks stand for significant differences between the wild type and the *pep2-1* mutant at each time point determined by multiple pairwise Bonferroni tests (α-value of 0.05). Error bars indicate the standard deviation.

To test the *PEP1*-dependent role of *PEP2*, we exposed the *pep2-1* mutant and the wild-type plants to different durations of vernalization. Both genotypes were grown for 5 weeks in LDs, vernalized for 8, 12, 18, and 21 weeks, and transferred back to LD greenhouse conditions (Fig. 5A, B). The *pep2-1* mutant showed a reduction in the number of days to flower emergence compared with the wild type in all durations of cold (Fig. 5C). In addition, inflorescences in *pep2-1* showed reduced floral reversion phenotypes and enhanced commitment of inflorescence branches to flowering (Fig. 5D–G). These results indicate that *PEP2* controls flowering time and inflorescence architecture in *A. alpina*. However, the response of *pep2-1* still varied with the duration of vernalization, suggesting that other floral repressors might contribute to flowering in response to vernalization. Also, *PEP2* is required to maintain axillary shoots that are located just below the inflorescence in a vegetative state as all axillary branches in the *pep2-1* mutant commit to reproductive development (Fig. 5; Bergonzi *et al.*, 2013).

**Fig. 6.**
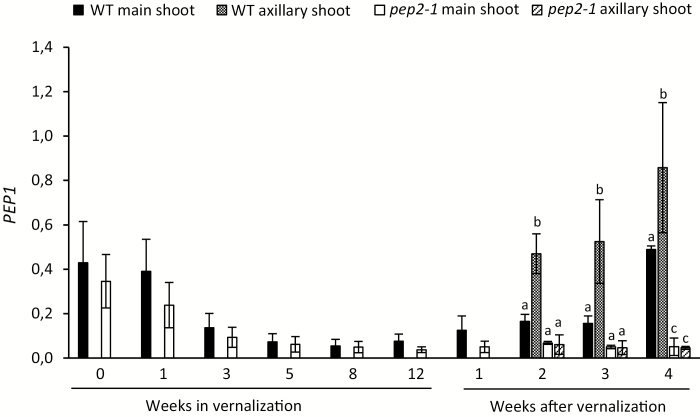
*PEP2* is required to activate *PEP1* expression after vernalization. Relative expression of *PEP1* in the shoot apical meristem and in the vegetative axillary meristems of the wild type (WT) and the *pep2-1* mutant before, during, and after 12 weeks of vernalization. Letters stand for significant differences between the WT and *pep2-1* at each time point determined by multiple pairwise comparisons using Benjamini–Hochberg-corrected *P*-values (α-value of 0.05). Detailed information on significant differences can be found in Supplementary Table S3. Error bars indicate the standard deviation.

As shown previously in the wild type, *PEP1* mRNA is up-regulated in the shoot apical meristem of the main shoot after a non-saturating vernalization (Fig. 6; Wang *et al.*, 2009; Lazaro *et al.*, 2018). This unstable silencing of *PEP1* mRNA after cold was abolished in the *pep2-1* mutant, suggesting that *PEP2* is required to activate *PEP1* expression in the shoot apical meristem after insufficient vernalization (Fig. 6). The role of *PEP2* in the activation of *PEP1* after vernalization is also observed in the axillary branches. All axillary branches in the *pep2-1* mutant committed to flowering (Fig. 5B) and showed very low expression of *PEP1* when compared with wild-type vegetative branches (Fig. 6). These results suggest that the major contribution of *PEP2* is to activate *PEP1* transcription after vernalization, both in the shoot apical meristem and in the vegetative axillary branches.

## Discussion

Understanding the role of prolonged exposure to low temperatures in flowering is of particular importance in perennial species that will overwinter several times during their life cycle. In temperate perennials, prolonged exposure to cold controls later stages of flowering such as uniform bud break in the spring, whereas in alpine species it ensures floral formation before plants experience favorable environmental conditions for anthesis (Diggle, 1997; Meloche and Diggle, 2001; Lazaro *et al.*, 2018). The maintenance of vegetative development after flowering, which is important for the perennial life strategy, is regulated by the seasonal cycling of floral repressors and the differential response of meristems to flower inductive stimuli due to age-related factors (Wang *et al.*, 2009, 2011; Koskela *et al.*, 2012). Here we characterized the role of the *A. alpina* floral repressor *PEP2*, the ortholog of the Arabidopsis *AP2.* Previous studies had demonstrated that *PEP2* controls flowering through a *PEP1*-dependent and a *PEP1*-independent pathway (Bergonzi *et al*., 2013). Our transcriptomic analysis indicated that *PEP2* influences the expression of genes involved in several developmental processes. However, many of the identified genes might not be regulated directly by PEP2 but by complex downstream genetic interactions (Fig. 1; Supplementary Fig. S1). We also found both floral promoters and repressors differentially expressed in the *pep2* mutant. In Arabidopsis, the AP2 protein was immunoprecipitated from the promoter region of *SOC1* (Yant *et al.*, 2010). However, in our study, the effect of *PEP2* on *AaSOC1* seems to be through *PEP1* (Fig. 1). To characterize the *PEP1*-dependent and the *PEP1*-independent role of *PEP2* in flowering, we also employed physiological analysis and followed the expression of flowering time and meristem identity genes during the *A. alpina* life cycle. These data indicated that *PEP2* controls (i) the age-dependent response to vernalization and (ii) the temporal cycling of the floral repressor *PEP1* by ensuring the activation of *PEP1* expression after vernalization.

### *PEP2* controls the age-dependent response to vernalization

*PEP2* could rescue the early flowering phenotype of the Arabidopsis *ap2-7* mutant, suggesting that its role in flowering time might be conserved (Fig. 2). In Arabidopsis, *AP2* is post-transcriptionally regulated by miR172, and *miR172b* is placed in the age pathway as it is transcriptionally controlled by the miR156 targets SPL9 and SPL15 (Wu *et al.*, 2009; Hyun *et al.*, 2016). AP2 also negatively regulates its own expression by directly binding to its own genomic locus, as well as to the loci of its regulators *miR156e*, *miR172b*, and *FUL*, suggesting that *AP2* is transcriptionally regulated by multiple feedback loops (Schwab *et al.*, 2005; Yant *et al.*, 2010; Balanza *et al*., 2018). The AP2 protein was also immunoprecipitated from the chromatin of floral integrators and genes required for floral meristem development such as *SOC1*, *AGAMOUS* (*AG*), and *AP1* (Yant *et al.*, 2010). The transcription of genes such as *SOC1* and *FUL* is also controlled by upstream regulators in the age pathway. SPL9 has been reported to bind to the first intron of *SOC1* and SPL15 to *FUL* and *miR172b* (Wang *et al.*, 2009; Hyun *et al.*, 2016). Overall, this complex genetic circuit that includes AP2 might contribute to the fast life cycle of Arabidopsis, in which floral transition takes place soon after reproductive competence is acquired. In contrast to Arabidopsis, in *A. alpina* reproductive competence is uncoupled from flowering initiation. *Arabis alpina* plants become competent to flower after growing for 5 weeks in LD conditions but only initiate flowering when they are exposed to vernalization (Wang *et al.*, 2009). This suggests that flowering in *A. alpina* is regulated by a strong interplay between the age and the vernalization pathways. Members of the SPL and AP2 families (e.g. *AaSPL15* and *AaTOE2*) are transcriptionally repressed by PEP1 in addition to the post-transcriptional and post-translational regulation by the miRNAs (Chen, 2004; Bergonzi *et al*., 2013; Hyun *et al.*, 2016; Xu *et al.*, 2016; Mateos *et al.*, 2017). Although, FLC in Arabidopsis targets a similar set of genes, the strong interplay between the age and the vernalization pathway is most apparent in *A. alpina* (Deng *et al.*, 2011; Mateos *et al.*, 2017). Vernalization in *A. alpina* provides the condition where the age effect on flowering is apparent as it silences *PEP1*. Gradual changes in the accumulation of miR156 and the expression of the *SPL* genes can be observed in the shoot apex of *A. alpina* plants that get older in LDs (Bergonzi *et al*., 2013). However, the accumulation of downstream regulators in the age pathway, such as of miR172, only increases in the shoot apex during vernalization and upon floral transition (Bergonzi *et al*., 2013). Here we show that the expression of miR156 and of *AaSPL5* and *15* is not influenced in *pep2* plants grown in LDs (Fig. 1E–G; Supplementary Fig. S3). Given that *PEP2* acts partially through *PEP1*, the lack of an effect in *pep2-1* on *AaSPL15* can be due either to the residual *PEP1* expression in the *pep2-1* mutant or to the existence of compensatory genetic mechanisms. Interestingly, *AaSPL9* mRNA levels were reduced in 6-week-old *pep2-1* seedlings compared with the wild type (Supplementary Fig. S3). This effect of *PEP2* on *AaSPL9*, though, cannot be explained by the feedback loops described in Arabidopsis as *AaSPL9* transcript levels would be expected to be higher in *pep2-1* than in the wild type (Supplementary Fig. S3; Yant *et al.*, 2010).

The *A. alpina* ortholog of *TFL1* (*AaTFL1*) has been previously reported to influence the effect of vernalization in an age-dependent manner, although its expression pattern does not differ between juvenile and adult apices before vernalization (Wang *et al.*, 2011). Here we show that vernalization accelerated flowering in young *pep2-1* seedlings compared with *pep1-1*, suggesting that *PEP2* also regulates the age-dependent response to vernalization in a *PEP1*-independent pathway (Fig. 3). Interestingly, in our RNAseq analysis, *AaTFL1* transcripts were reduced in the *pep2-1* mutant, suggesting that *PEP2*, together with or through *AaTFL1*, sets an age threshold for flowering in response to vernalization. One major difference between *AaTFL1* and *PEP2*, though, is that lines with reduced *AaTFL1* activity do not flower without vernalization. These results suggest that *PEP2* plays additional roles in the regulation of flowering time in *A. alpina*. Transcriptomic experiments in Arabidopsis also showed that *TFL1* mRNA is down-regulated in *ap2* inflorescences compared with the wild type (Yant *et al.*, 2010). However, no direct binding of AP2 to the *TFL1* locus has been detected by ChIP-Seq and therefore it is unclear whether there is a direct or indirect effect of AP2 on *TFL1* transcription (Yant *et al.*, 2010).

### *PEP2* ensures the activation of *PEP1* after vernalization

Previous studies in *A. alpina* have demonstrated that *PEP2* controls flowering in response to vernalization via enhancing the expression of *PEP1* (Bergonzi *et al*., 2013). Here we show that the major role of *PEP2* in *PEP1* activation takes place after vernalization. *PEP1* expression in *A. alpina* is temporarily silenced during prolonged exposure to cold to define inflorescence fate, while it is highly expressed in axillary branches after vernalization to repress flowering (Wang *et al.*, 2009; Lazaro *et al.*, 2018). We have recently shown that the duration of vernalization influences *PEP1* reactivation in the shoot apex after the return to warm temperatures (Lazaro *et al.*, 2018). Phenotypes correlated with high *PEP1* mRNA levels after vernalization (e.g. floral reversion and the presence of vegetative axillary branches) were almost absent in the *pep2-1* mutant (Fig. 5; Lazaro *et al.*, 2018). Accordingly, *PEP1* mRNA levels were reduced in vernalized *pep2-1* plants compared with the wild type both in the inflorescence stem and in the axillary branches (Fig. 6; Wang *et al.*, 2009; Lazaro *et al.*, 2018). These results suggest that *PEP2* contributes to the perennial life cycle and controls perennial-specific traits via activating *PEP1* after vernalization. In Arabidopsis, the introgression of the *FRI* allele from the Sf-2 accession into Col extends the duration of cold temperatures required to silence *FLC* (Searle *et al.*, 2006). Northern Arabidopsis accessions such as Lov-1 require several months of vernalization to achieve *FLC* silencing and, similarly to *A. alpina* Pajares, a shorter duration of cold temperatures causes *FLC* reactivation (Shindo *et al.*, 2006). The link between *AP2* and *FLC* is not clear in Arabidopsis. AP2 does not bind to the *FLC* locus in ChIP-Seq experiments, and in our study *FLC* expression was not altered in plants where the *ap2-7* mutant allele was introgressed into the Col *FRI* Sf-2 background (Supplementary Fig. S3; Yant *et al.*, 2010). However, as the strongest difference in *PEP1* expression in the *pep2-1* mutant was observed after vernalization, the effect of *AP2* in the Lov-1 accession should be analyzed to rule out a role for *AP2* on *FLC* reactivation after insufficient vernalization.

The unstable silencing of *FLC* involves changes in the accumulation of the H3 trimethylation at Lys27 (H3K27me3) (Angel *et al.*, 2011; Coustham *et al.*, 2012). The pattern of the H3K27me3 mark at the *PEP1* locus also correlates with changes in *PEP1* mRNA levels in *A. alpina* (Wang *et al.*, 2009). *PEP1* shows a much higher and broader increase of H3K27me3 during the cold than *FLC*, and H3K27me3 levels rapidly decrease at *PEP1* after short vernalization periods (Wang *et al.*, 2009; Angel *et al.*, 2011; Lazaro *et al.*, 2018). Although the proteins regulating histone modifications at the *PEP1* locus are not known, resetting of the epigenetic memory of *FLC* in Arabidopsis is dependent on the presence of TrxG components and the Jumonji C (JmjC) domain-containing demethylases EARLY FLOWERING 6 (ELF6) and RELATIVE OF EARLY FLOWERING 6 (REF6) (Noh *et al.*, 2004; Yun *et al.*, 2011; Crevillen *et al*., 2014). It has been shown that AP2 has the ability to interact with a chromatin remodeling factor HISTONE DEACETYLASE 19 (HDA19) to transcriptionally repress one of its targets (Krogan *et al.*, 2012), but AP2 has never been associated with histone demethylases.

We have recently demonstrated that *PEP1* is stably silenced in the shoot apical meristem of adult plants that commit to flowering during prolonged exposure to cold (Lazaro *et al.*, 2018). In juvenile plants, a similar duration of vernalization fails to initiate flowering even if *PEP1* is silenced during cold (Lazaro *et al.*, 2018). Floral commitment during vernalization is correlated with a higher expression of the floral meristem identity genes, *AaFUL*, *AaLFY*, and *AaAP1*, which are repressed by *PEP2* (Lazaro *et al.*, 2018). This is evident by the precocious up-regulation of *AaFUL*, *AaLFY*, and *AaAP1* mRNA levels in vernalized *pep2-1* plants compared with the wild type (Fig. 4; Supplementary Fig. S4). Although, the link between *PEP2* and *PEP1* resetting is not clear, it seems that the achievement of floral commitment during vernalization is negatively correlated with *PEP1* up-regulation after the return to warm temperatures (Lazaro *et al.*, 2018). In Arabidopsis, AP2 is not known to influence *FLC* transcription. However, *AP2* has been reported to be transcriptionally repressed by FUL, and *FUL*-overexpressing plants show reduced *FLC* expression (Balanzà *et al.*, 2014, 2018). These results suggest that FUL might regulate *FLC* transcription independently or through *AP2*. This might also indicate that in *A. alpina* the role of *PEP2* in *PEP1* expression might implicate other flowering time regulators, genes involved in the age pathway and genes ensuring floral commitment during vernalization. However, since PEP1 also transcriptionally regulates genes in these genetic pathways, feedback mechanisms might also occur (Mateos *et al.*, 2017).

### Conclusion

Our study demonstrates that *PEP2* plays an instrumental role in *A. alpina* controlling the age-dependent response to vernalization and facilitating the activation of *PEP1* after vernalization. As both roles of *PEP2* focus on whether floral commitment has been achieved during vernalization, suggests that they might not be completely independent. Upstream regulators of floral meristem identity genes such as PEP2 might control the response to vernalization of individual meristems and contribute to the complex plant architecture of perennials.

## Supplementary data

Supplementary data are available at *JXB* online.

**Dataset S1.** Transcripts identified as being differentially expressed in *pep2-1* compared with the wild type.

**Dataset S2.** Transcripts identified as being differentially expressed in *pep1-1* compared with the wild type.

**Dataset S3.** Transcripts identified as being differentially expressed in *pep2-1* compared with *pep1-1*.

**Fig. S1.** GO-enriched categories in the RNAseq experiment.

**Fig. S2.** *AP2* activity does not affect *FLC* expression in Arabidopsis.

**Fig. S3.** The expression levels of miR156, *AaSPL5*, and *AaSPL15* do not differ between wild-type and *pep2-1* plants growing in long days.

**Fig. S4.** *PEP2* regulates the age-dependent response of *A. alpina* to vernalization.

**Fig. S5.** *PEP2* controls *AaFUL*, *AaTFL1*, *AaLFY*, and *AaAP1* expression during vernalization in adult plants.

**Table S1.** Primers used for PIPE-cloning of the *PEP2* locus.

**Table S2.** Statistical differences in Supplementary Fig. S2 determined by multiple pairwise comparisons using Benjamini–Hochberg-corrected *P*-values comparing *FLC* mRNA levels between *FRI* and *FRI ap2-7* at different developmental stages.

**Table S3.** Statistical differences in Fig. 6 determined by multiple pairwise comparisons using Benjamini–Hochberg-corrected *P*-values comparing *PEP1* mRNA levels between *pep2-1* and the wild type at different developmental stages.

Supplementary_Dataset_S1

Supplementary_Dataset_S2

Supplementary_Dataset_S3

Supplementary_Figure_S1-S5

Supplementary_Table_S1-S3

## Abbreviations

DAG: days after germination
LD: long day
SD: short day.
