# Supplementary material for: PERPETUAL FLOWERING2 coordinates the vernalization response and perennial flowering in Arabis alpina
Source: J Exp Bot. 2018 Nov 27;70(3):949–61. doi: 10.1093/jxb/ery423 (PMC6363098; doi:10.1093/jxb/ery423)
Supplement: Supplementary_Figure_S1-S5 [file ery423_suppl_supplementary_figure_s1-s5.pdf]

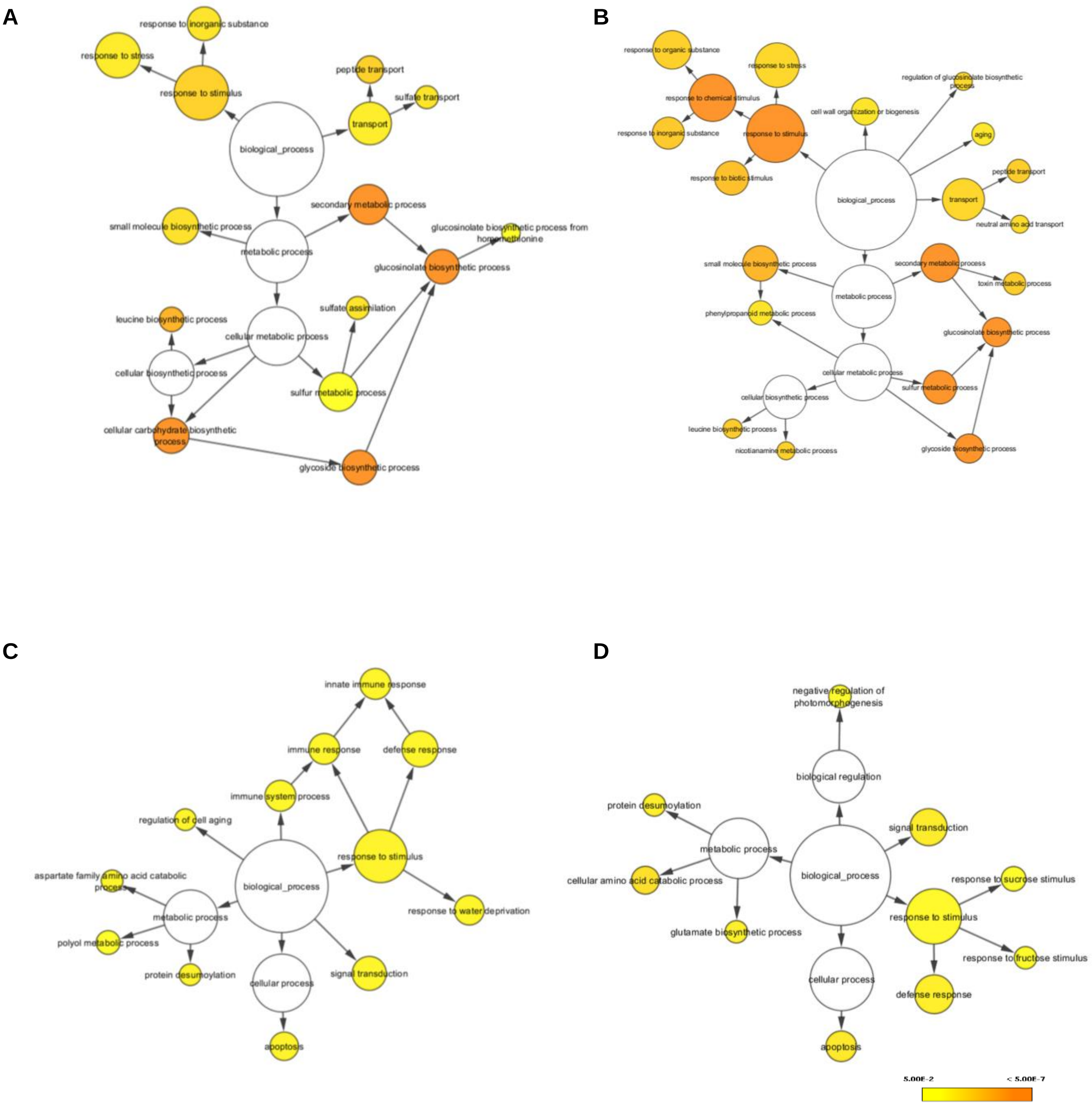

Fig. S1. GO enriched categories in RNAseq experiment. Bubble network shows GO terms enriched among differentially expressed genes in *pep2-1*. Color represents p value, and size of the bubble represents the representation factor. Hypergeometric test Benjamini–Hochberg FDR correction. Cutoff 0.05. **(A)** Up regulated in *pep2-1* compared to the wild type (WT). **(B)** Up regulated in *pep2-1* compared to *pep1-1*. **(C)** Down regulated in *pep2-1* compared to the WT. **(D)** Down-regulated in *pep2-1* compared to *pep1-1*.

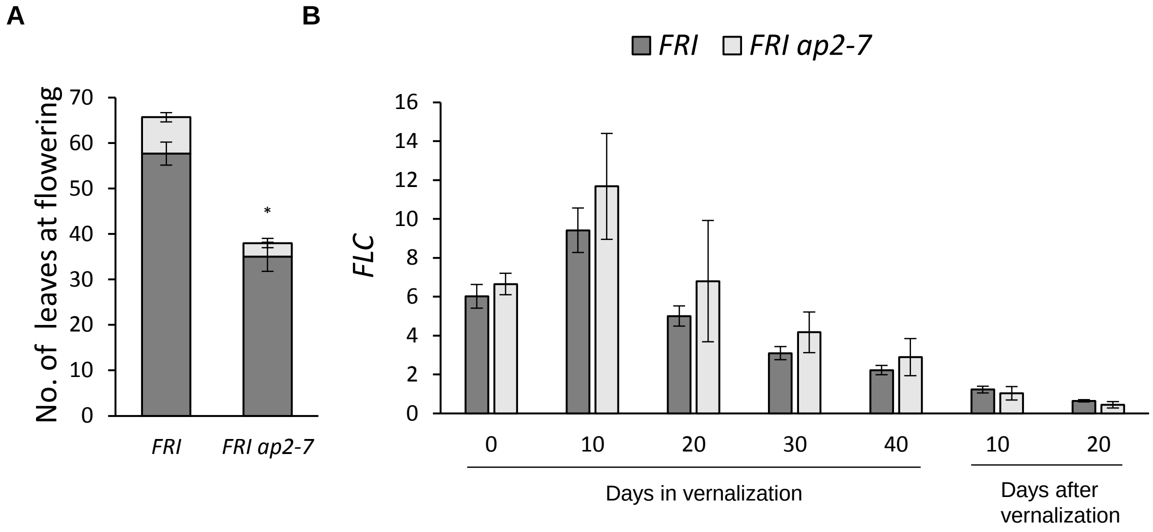

Fig. S2. *AP2* does not affect *FLC* expression in Arabidopsis. (A) Flowering time of *FRI* and *FRI ap2-7* plants scored as the number of leaves at flowering. Dark grey color represents rosette leaves and light grey color cauline leaves. The asterisk stands for a significant difference in the total leaf number determined by a Student T-test (p-value<0.01). **(B)** Relative expression of *FLC* in the shoot apical meristem of *FRI* and *FRI ap2-7* plants before, during and after 40 days of vernalization. There are no significant differences between *FRI* and *FRI ap2-7* samples at each time point determined by multiple pairwise comparisons using Benjamini-Hochberg-corrected p-values (α-value of 0.05). Detailed information on significant differences can be found in Table S2. Error bars indicate s.d.m.

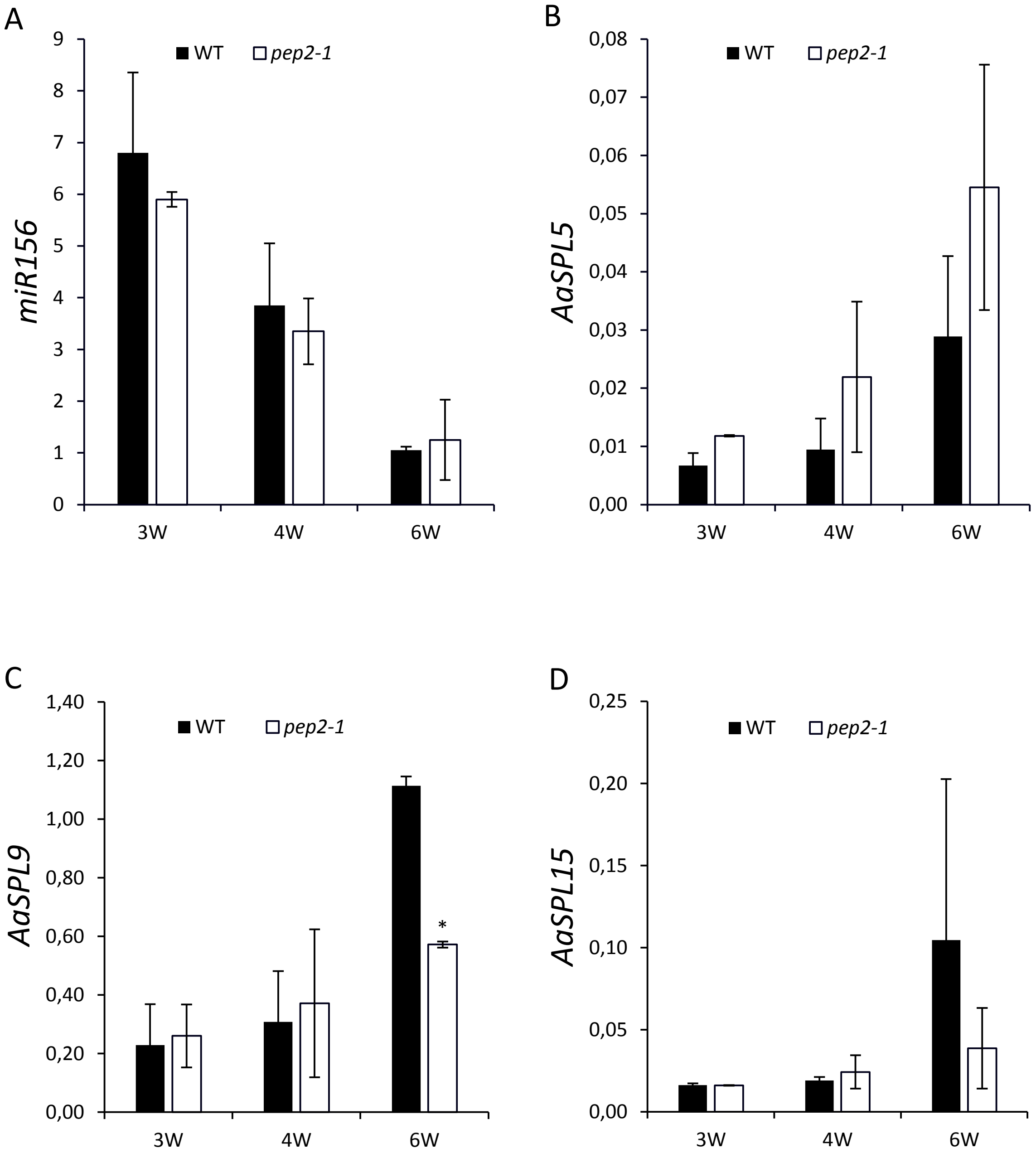

Fig. S3. The expression level of *miR156*, *AaSPL5* and *AaSPL15* does not differ between wild type and *pep2-1* plants growing in long days. Relative expression of miR156 **(A)**, *AaSPL5* **(B)***, AaSPL9* **(C)** and *AaSPL15* **(D)** in wild type (WT) and the *pep2-1* mutant. Apices were harvested from WT and *pep2-1* seedlings growing for 3, 4 and 6 weeks in LDs. Asterisks stand for significant differences between WT and *pep2-1* at each time point determined by multiple pairwise comparisons using Benjamini-Hochberg-corrected p-values (α-value of 0.05). Values are the average of 2 biological replicates, error bars indicate s.d.m.

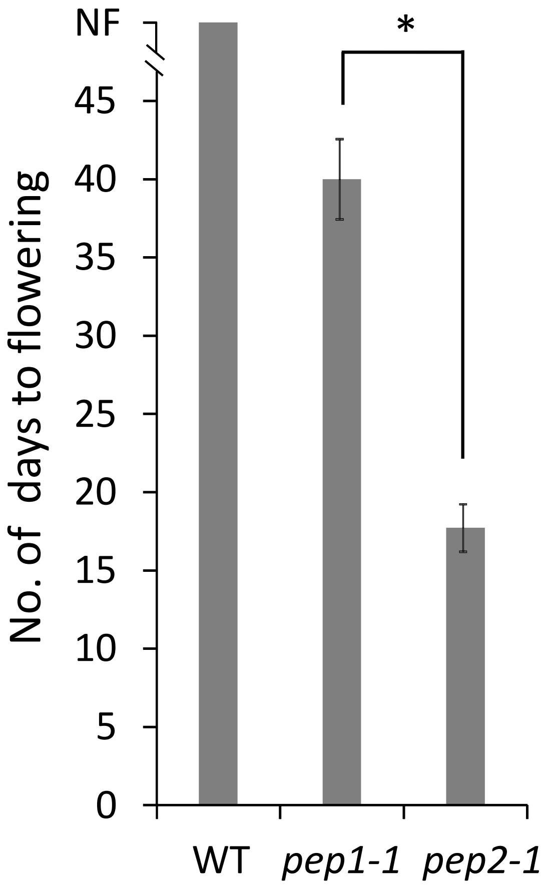

Fig. S4. *PEP2* regulates the age-dependent response of *A. alpina* to vernalization. Flowering time demonstrated as the number of days to flower emergence of 3-week-old wild type (WT), *pep1-1* mutant and *pep2-1* mutant, vernalized for 12 weeks. WT did not flower (NF). Error bars indicate s.d.m.

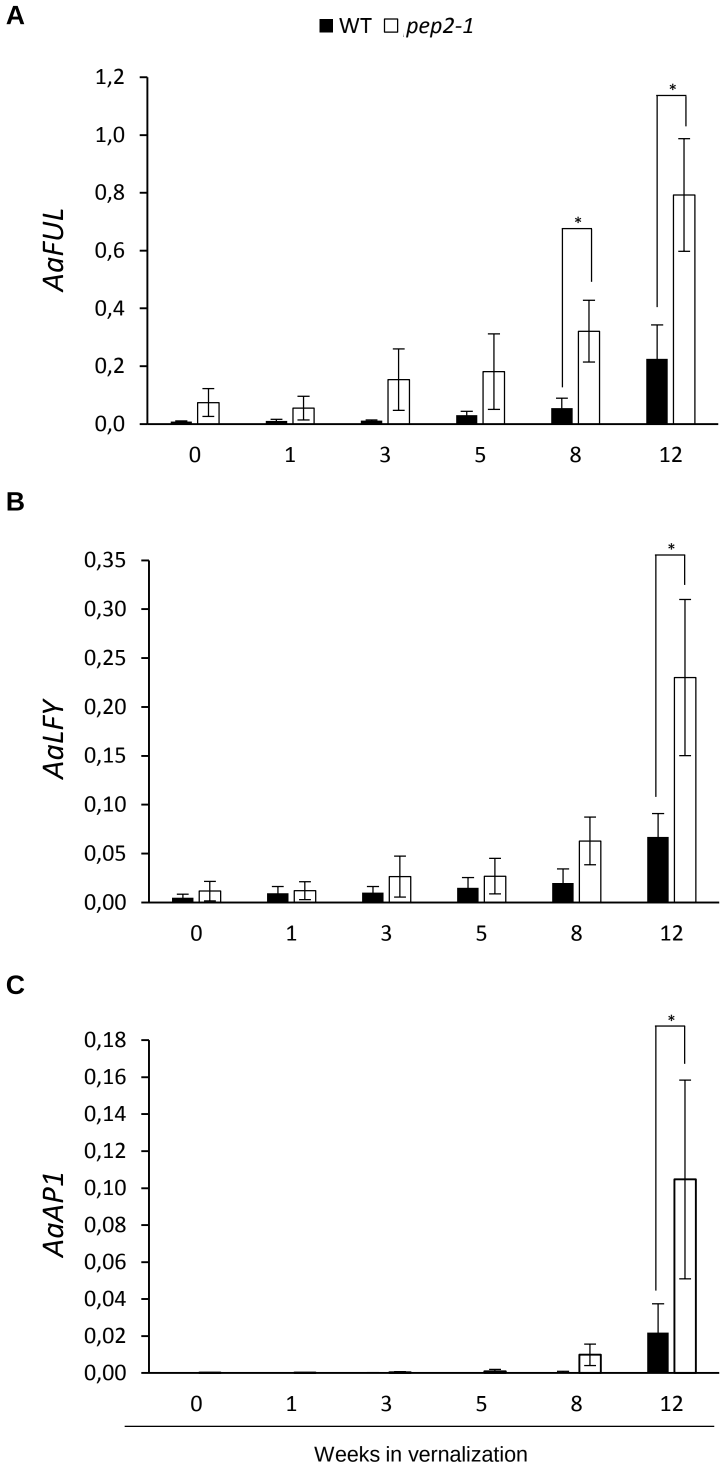

Fig. S4. ***PEP2*** **regulates** ***AaFUL*****,** ***AaTFL1*****,** ***AaLFY*** **and** ***AaAP1*** **expression during vernalization in adult plants.** Relative expression of *AaFUL* **(A)**, *AaLFY* **(B)**, *AaAP1* **(C).** 6-week-old wild type (WT) and *pep2-1* shoot apices were harvested before and during 12 weeks of vernalization. Asterisks stand for significant differences between the WT and *pep2-1* at each time point determined by multiple pairwise comparisons using Benjamini-Hochberg-corrected p-values (α-value of 0.05). Error bars indicate s.d.m.
