# Supplementary material for: PERPETUAL FLOWERING2 coordinates the vernalization response and perennial flowering in Arabis alpina
Source: J Exp Bot. 2018 Nov 27;70(3):949–61. doi: 10.1093/jxb/ery423 (PMC6363098; doi:10.1093/jxb/ery423)
Supplement: Supplementary_Table_S1-S3 [file ery423_suppl_supplementary_table_s1-s3.pdf]

Table S1. Primers used for PIPE-cloning of the *PEP2* locus

PEP2 4Kb promoter region cloning	Forward	GGGGACAAGTTTGTACAAAAAAGCAGGCTTCGTCCAGATCCCGGACATGGC
	Reverse	TGTGGTGCATCATTTAGATCCCACATTTTTTTTTTTTTGGTTATAAAAAGTTTTAAGAAAATTTCAATCT
PEP2 from ATG to the downstream gene	Forward	GGGGACAAGTTTGTACAAAAAAGCAGGCTTCATGTGGGATCTAAATGATGCACCACA
	Reverse	GGGGACCACTTTGTACAAGAAAGCTGGGTCCTAGCATTACTATGATTTTATATATATAGAGGAGATCTTGCGG
PIPE for *pPEP2*::*PEP2*	Forward	AGATTGAAATTTTCTTAAAACTTTTTATAACCAAAAAAAAAAAAATGTGGGATCTAAATGATGCACCACA
	Reverse	GAAGCCTGCTTTTTTGTACAAACTTGGC
C-terminal VENUS-9Ala cloning	Forward	TCCAACCTCCTCTCATGAGACCTTCTATGGCTGCGGCTGCTGC
	Reverse	CTTTTTCAGAATTTGTCACTTATATATATATAAAAAGGGATTTAGAATAATAAAGATTTACTTGTACAGCTCGTCCATGCCG
PIPE for *pPEP2*::*PEP2*::*VENUS-9Ala*	Forward	GCAGCAGCCGCAGCCATAGAAGGTCTCATGAGAGGAGGTTGGA
	Reverse	CGGCATGGACGAGCTGTACAAGTAAATCTTTATTATTCTAAATCCCTTTTTATATATATATAAGTGACAAATTCTGAAAAAG
N-terminal VENUS-9Ala cloning	Forward	GATAGATTGAAATTTTCTTAAAACTTTTTATAACCAAAAAAAAAAAAATGGTGAGCAAGGGCGAGG
	Reverse	CGCTGCCGCAGCGGCAGCAGCCGCAGCGCCCTTGTACAGCTCGTCCATGCCG
PIPE for *pPEP2::VENUS-9Ala::PEP2*	Forward	TGCCGCTGCGGCAGCGATGTGGGATCTAAATGATGCACCACA
	Reverse	CCTCGCCCTTGCTCACCATTTTTTTTTTTTTGGTTATAAAAAGTTTTAAGAAAATTTCAATCTATC

Table S2 Statistical differences in Figure S2 determined by multiple pairwise comparisons using Benjamini-Hochberg-corrected p-values comparing *FLC* mRNA levels between *FRI* and *FRI ap2-7* at different developmental stages. The comparisons highlighted in yellow are significantly different (α-value of 0.05).

	ap2 0	ap2 10	ap2 20	ap2 30	ap2 40	ap2 10AV	ap2 20AV	FRI 0	FRI 10	FRI 20	FRI 30	FRI 40	FRI 10AV
ap2 10	0.00388	-	-	-	-	-	-	-	-	-	-	-	-
ap2 20	0.90625	0.0046	-	-	-	-	-	-	-	-	-	-	-
ap2 30	0.10564	0.00024	0.08783	-	-	-	-	-	-	-	-	-	-
ap2 40	0.01998	0.000067	0.01696	0.39686	-	-	-	-	-	-	-	-	-
ap2 10AV	0.0019	0.000018	0.00167	0.04615	0.21223	-	-	-	-	-	-	-	-
ap2 20AV	0.001	0.000018	0.0009	0.02032	0.10624	0.68568	-	-	-	-	-	-	-
FRI 0	0.67245	0.0019	0.60086	0.21316	0.04624	0.00391	0.0019	-	-	-	-	-	-
FRI 10	0.07517	0.13771	0.08896	0.00285	0.00077	0.000087	0.000067	0.03201	-	-	-	-	-
FRI 20	0.26256	0.00066	0.22518	0.58313	0.16414	0.01552	0.00676	0.50678	0.00831	-	-	-	-
FRI 30	0.02578	0.000076	0.0208	0.48316	0.88647	0.17151	0.08564	0.06083	0.00091	0.20651	-	-	-
FRI 40	0.00824	0.000046	0.00676	0.19981	0.65729	0.42811	0.22518	0.01969	0.00034	0.07402	0.57001	-	-
FRI 10AV	0.0023	0.000018	0.0019	0.06002	0.25823	0.88647	0.60086	0.00501	0.0001	0.01969	0.21223	0.50792	-
FRI 20AV	0.00123	0.000018	0.00103	0.02634	0.13771	0.79724	0.88647	0.00239	0.000067	0.00863	0.10624	0.28038	0.68568

Table S3. Statistical differences in Figure 6 determined by multiple pairwise comparisons using Benjamini-Hochberg-corrected p-values comparing *PEP1* mRNA levels between *pep2-1* and the wild type at different developmental stages. The comparisons highlighted in yellow are significantly different (α-value of 0.05).

	WT 0	WT 1	WT 12	WT 3	WT 5	WT 8	WT 1AV	WT 2AV	WT 3AV	WT 4AV	WT ax2AV	WT ax3AV	WT ax4AV	pep2 0	pep2 1	pep2 12	pep2 3	pep2 5	pep2 8	pep2 1AV	pep2 2AV	pep2 3AV	pep2 4AV	pep2 ax2AV	pep2 ax3AV
WT 1	0.83455	-	-	-	-	-	-	-	-	-	-	-	-	-	-	-	-	-	-	-	-	-	-	-	-
WT 12	8.0e-05	0.00031	-	-	-	-	-	-	-	-	-	-	-	-	-	-	-	-	-	-	-	-	-	-	-
WT 3	0.00065	0.00286	0.62382	-	-	-	-	-	-	-	-	-	-	-	-	-	-	-	-	-	-	-	-	-	-
WT5	7.2e-05	0.00028	0.99248	0.59535	-	-	-	-	-	-	-	-	-	-	-	-	-	-	-	-	-	-	-	-	-
WT 8	3.9e-05	0.00014	0.97189	0.45528	0.99248	-	-	-	-	-	-	-	-	-	-	-	-	-	-	-	-	-	-	-	-
WT 1AV	0.00045	0.00188	0.74398	0.99248	0.70717	0.53482	-	-	-	-	-	-	-	-	-	-	-	-	-	-	-	-	-	-	-
WT 2AV	0.00559	0.01979	0.46289	0.94627	0.44588	0.34318	0.85910	-	-	-	-	-	-	-	-	-	-	-	-	-	-	-	-	-	-
WT 3AV	0.00140	0.00592	0.46289	0.98281	0.44588	0.33095	0.91479	0.99248	-	-	-	-	-	-	-	-	-	-	-	-	-	-	-	-	-
WT 4AV	0.69628	0.41706	4.7e-05	0.00030	4.2e-05	2.9e-05	0.00020	0.00196	0.00057	-	-	-	-	-	-	-	-	-	-	-	-	-	-	-	-
WT ax2AV	0.85910	0.53052	8.2e-05	0.00057	7.7e-05	4.3e-05	0.00039	0.00349	0.00105	0.99248	-	-	-	-	-	-	-	-	-	-	-	-	-	-	-
WT ax3AV	0.43342	0.21899	2.0e-05	9.8e-05	1.8e-05	9.9e-06	7.1e-05	0.00065	0.00017	0.93224	0.79045	-	-	-	-	-	-	-	-	-	-	-	-	-	-
WT ax4AV	3.3e-05	1.0e-05	1.3e-10	6.5e-10	1.3e-10	9.3e-11	5.0e-10	1.9e-08	1.3e-09	0.00051	0.00028	0.00148	-	-	-	-	-	-	-	-	-	-	-	-	-
pep2 0	0.44157	0.79045	0.00163	0.01481	0.00144	0.00071	0.00981	0.06880	0.02858	0.17787	0.27184	0.07129	2.2e-06	-	-	-	-	-	-	-	-	-	-	-	-
pep2 1	0.02764	0.08959	0.06783	0.32335	0.06121	0.03490	0.25702	0.58062	0.44588	0.00872	0.01607	0.00267	2.9e-08	0.29488	-	-	-	-	-	-	-	-	-	-	-
pep2 12	2.6e-05	8.0e-05	0.83525	0.34318	0.86276	0.99248	0.42173	0.25099	0.22861	1.8e-05	2.9e-05	5.8e-06	9.3e-11	0.00039	0.01979	-	-	-	-	-	-	-	-	-	-
pep2 3	0.00014	0.00059	0.99248	0.79723	0.97746	0.83771	0.90839	0.58550	0.60115	7.9e-05	0.00014	3.0e-05	2.2e-10	0.00302	0.11010	0.66497	-	-	-	-	-	-	-	-	-
pep2 5	4.9e-05	0.00018	0.99248	0.49843	0.99248	0.99248	0.59535	0.37887	0.37457	3.3e-05	5.4e-05	1.2e-05	1.0e-10	0.00093	0.04375	0.96159	0.90839	-	-	-	-	-	-	-	-
pep2 8	3.6e-05	0.00012	0.94627	0.42706	0.96173	0.99248	0.49618	0.31634	0.30275	2.6e-05	3.9e-05	8.6e-06	9.3e-11	0.00060	0.02968	0.99248	0.79555	0.99248	-	-	-	-	-	-	-
pep2 1AV	3.6e-05	0.00012	0.94627	0.43065	0.96173	0.99248	0.49843	0.31855	0.30556	2.6e-05	3.9e-05	8.6e-06	9.3e-11	0.00061	0.03014	0.99248	0.79723	0.99248	0.99660	-	-	-	-	-	-
pep2 2AV	0.00022	0.00079	0.99248	0.61402	0.99248	0.99248	0.71828	0.46289	0.47014	0.00011	0.00018	4.2e-05	6.5e-10	0.00349	0.08935	0.94627	0.96173	0.99248	0.99248	0.99248	-	-	-	-	-
pep2 3AV	3.6e-05	0.00012	0.94627	0.43035	0.96173	0.99248	0.49843	0.31854	0.30534	2.6e-05	3.9e-05	8.6e-06	9.3e-11	0.00061	0.03007	0.99248	0.79723	0.99248	0.99660	0.99697	0.99248	-	-	-	-
pep2 4AV	0.00013	0.00047	0.96173	0.49081	0.98281	0.99248	0.57369	0.37531	0.37531	7.1e-05	0.00012	2.9e-05	4.8e-10	0.00199	0.05773	0.99248	0.84326	0.99248	0.99660	0.99660	0.99248	0.99660	-	-	-
pep2 ax2AV	0.00017	0.00063	0.99248	0.56190	0.99248	0.99248	0.65322	0.43342	0.43425	9.1e-05	0.00015	3.6e-05	5.8e-10	0.00280	0.07395	0.97279	0.93224	0.99660	0.99248	0.99248	0.99248	0.99248	0.99248	-	-
pep2 ax3AV	0.00011	0.00040	0.94627	0.46175	0.96173	0.99248	0.53482	0.34976	0.34704	6.2e-05	0.00010	2.7e-05	4.4e-10	0.00173	0.05089	0.99248	0.80363	0.99248	0.99527	0.99248	0.99248	0.99248	0.99248	0.99248	-
pep2 ax4AV	0.00011	0.00039	0.94627	0.45769	0.96159	0.99248	0.53239	0.34704	0.34569	6.1e-05	0.00010	2.7e-05	4.4e-10	0.00170	0.05016	0.99248	0.79861	0.99248	0.99248	0.99248	0.99248	0.99248	0.99248	0.99248	0.99660
